# Introduction

**Published:** 1852-04-01

**Authors:** Francis Barlow

**Affiliations:** one of the Masters in Lunacy, and a Special Jury


### IMPORTANT LUNACY CASE

OF

### MRS. CATHERINE CUMMING,

*Tried before* FRANCIS BARLOW, Esq., *one of the Masters in Lunacy, and a Special Jury,*

AT THE EYRE ARMS, ST. JOHN'S WOOD,

January 7th to 24th, inclusive,

1852.

